# Posttraumatic Long-Segment Bulbar Urethral Stricture Treated With Perineal End-to-End Urethroplasty: A Case Report

**DOI:** 10.7759/cureus.88203

**Published:** 2025-07-17

**Authors:** Stanislaw Szymkiewicz

**Affiliations:** 1 Department of Urology, Janusz Korczak Provincial Specialist Hospital in Słupsk, Slupsk, POL

**Keywords:** antegrade urethrography, bulbar urethral stricture, end-to-end urethroplasty, pelvic fracture, suprapubic cystostomy

## Abstract

Posttraumatic urethral strictures following pelvic fractures represent a complex urological challenge that requires precise imaging, careful surgical planning, and evidence-based perioperative care. We present the case of a 45‑year‑old male patient with a suprapubic cystostomy placed one year earlier after a pelvic fracture, admitted for definitive management of urinary retention caused by a complete obstruction of the bulbar urethra. Antegrade urethrography revealed a long‑segment bulbar obliteration of approximately 3-5 cm. Surgical treatment consisted of perineal end‑to‑end urethroplasty over a 16 Ch Foley catheter using interrupted 5‑0 Monocryl sutures. A single preoperative dose of IV gentamicin (5 mg/kg) was administered 60 minutes before skin incision. Postoperatively, the Foley catheter was maintained for 14 days; enoxaparin 4,000 IU was given daily during hospitalization for thromboprophylaxis; and trimethoprim 100 mg was prescribed nightly for 14 days. Supportive care included nonsteroidal anti-inflammatory drugs (NSAIDs)/acetaminophen for analgesia, hydration of 2-3 L/day, and activity restriction for 4-6 weeks. On postoperative day 2, the patient was discharged asymptomatic with good perineal wound healing and a well‑positioned suprapubic catheter. He was scheduled for catheter removal and outpatient assessment after 14 days. The patient provided written informed consent for publication. This case underscores the importance of antegrade imaging for surgical planning, meticulous technique for tension‑free anastomosis, and perioperative protocols that collectively optimize outcomes in complex urethral reconstruction.

## Introduction

Urethral strictures secondary to pelvic trauma often result in complex long‑segment obliterations that challenge urologists [1]. Reliable imaging, such as combined antegrade and retrograde urethrography, is essential for defining stricture length and site, shaping operative strategy [2]. While longer strictures sometimes necessitate grafting or staged repair, appropriately selected cases benefit from end‑to‑end reconstruction [3,4]. Here, we highlight a case featuring complete bulbar urethral obliteration successfully managed with a tension‑free perineal anastomotic urethroplasty guided by careful imaging and perioperative management.

## Case presentation

**Figure 1:**
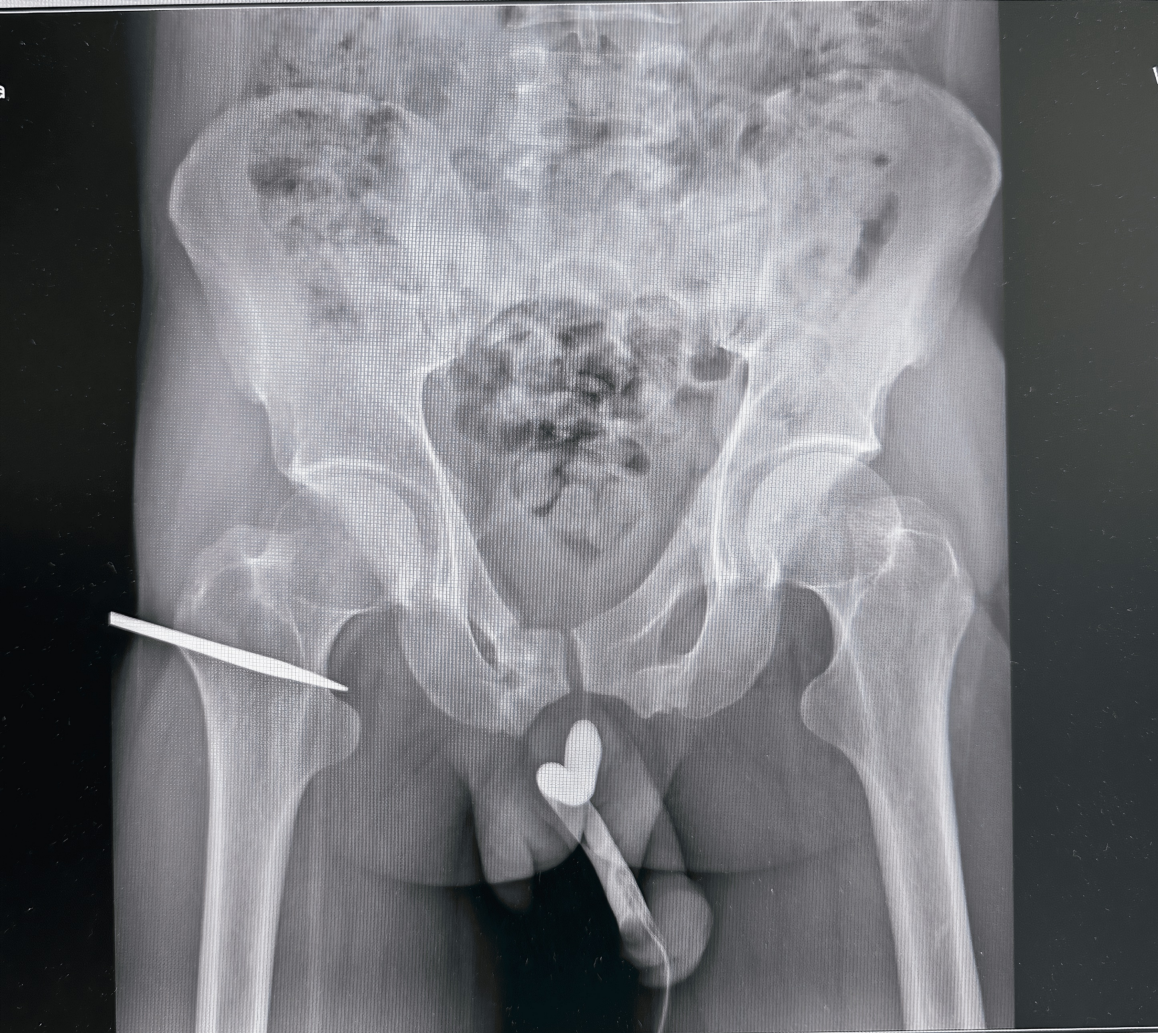
Retrograde urethrography demonstrating complete discontinuity of the bulbar urethra with no distal contrast opacification-typical appearance of posttraumatic obliteration

**Figure 2:**
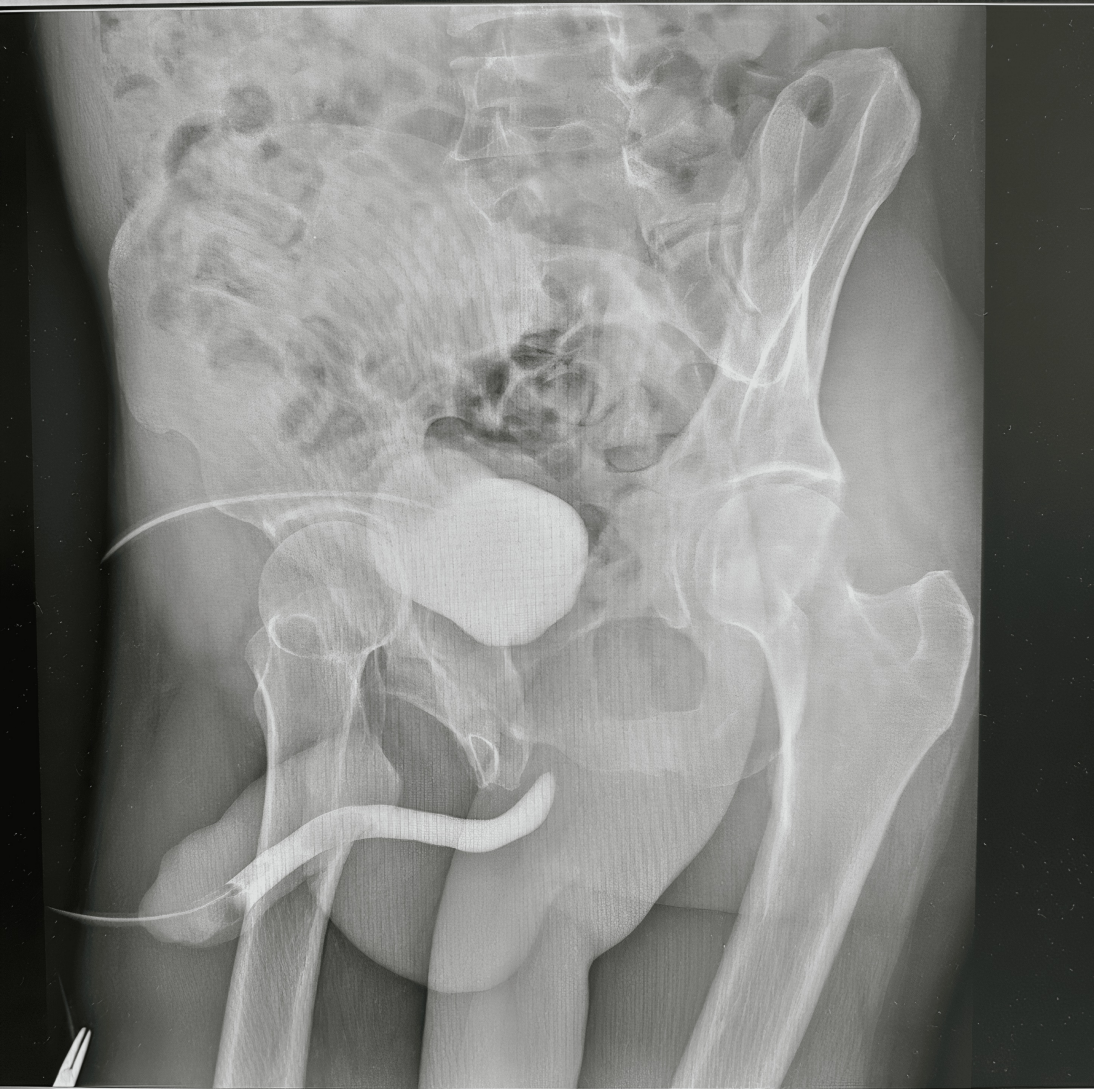
Antegrade urethrogram via suprapubic catheter shows a 3-5 cm bulbar urethral obliteration and retrograde urethrography demonstrating complete discontinuity of the bulbar urethra

A 45‑year‑old man with a history of pelvic fracture one year prior and a suprapubic catheter presenting for urinary retention was referred to our center for complex urethral reconstruction. Laboratory tests showed normal renal function (creatinine 1.1 mg/dL, estimated glomerular filtration rate (eGFR) 85 mL/min), balanced electrolytes, normal blood counts, and standard coagulation parameters. Retrograde urethrography (Figure 1) revealed complete discontinuity of a 3-5 cm segment of the bulbar urethra, which was confirmed by antegrade imaging via the suprapubic catheter (Figure 2), forming the basis for planning end-to-end repair.

Therapeutic intervention

**Figure 3:**
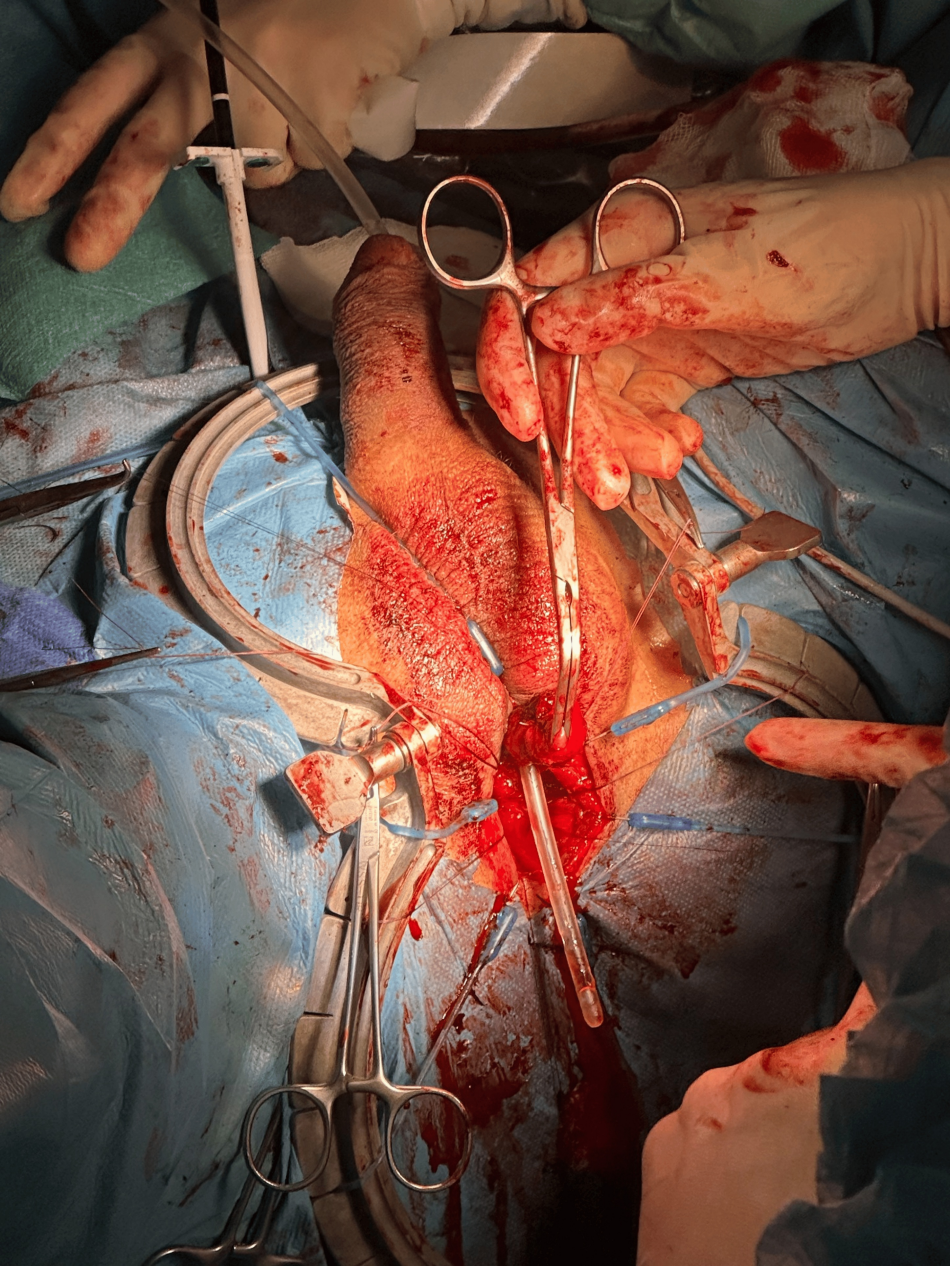
Intraoperative view after fibrotic segment excision preparing for end‑to‑end anastomosis

Preoperatively, IV gentamicin (5 mg/kg) was administered 60 minutes before incision, in keeping with prophylactic guidelines, to target Gram‑negative organisms known to complicate genitourinary procedures. Under general anesthesia in lithotomy position, the perineal region was prepped and draped. A midline incision exposed the bulbous urethra. The strictured fibrotic segment was visualized with cystofibroscopy via the suprapubic tract and then excised. Mobilization of both urethral ends and spatulation prepared the site for tension‑free anastomosis over a 16 Ch Foley catheter using interrupted 5‑0 Monocryl sutures (Figure 3). Enoxaparin 4,000 IU daily was initiated 12 hours postoperatively for thromboembolism prevention throughout the hospital stay. Additionally, trimethoprim 100 mg nightly was prescribed for 14 days to reduce the risk of catheter-associated urinary infections. The patient received multimodal analgesia (nonsteroidal anti-inflammatory drugs (NSAIDs), acetaminophen), was encouraged to drink 2-3 L of fluids daily, and was advised to abstain from strenuous activity for 4-6 weeks. A follow‑up appointment for catheter removal and outpatient evaluation was scheduled on day 14.

Follow‑up and outcomes

**Figure 4:**
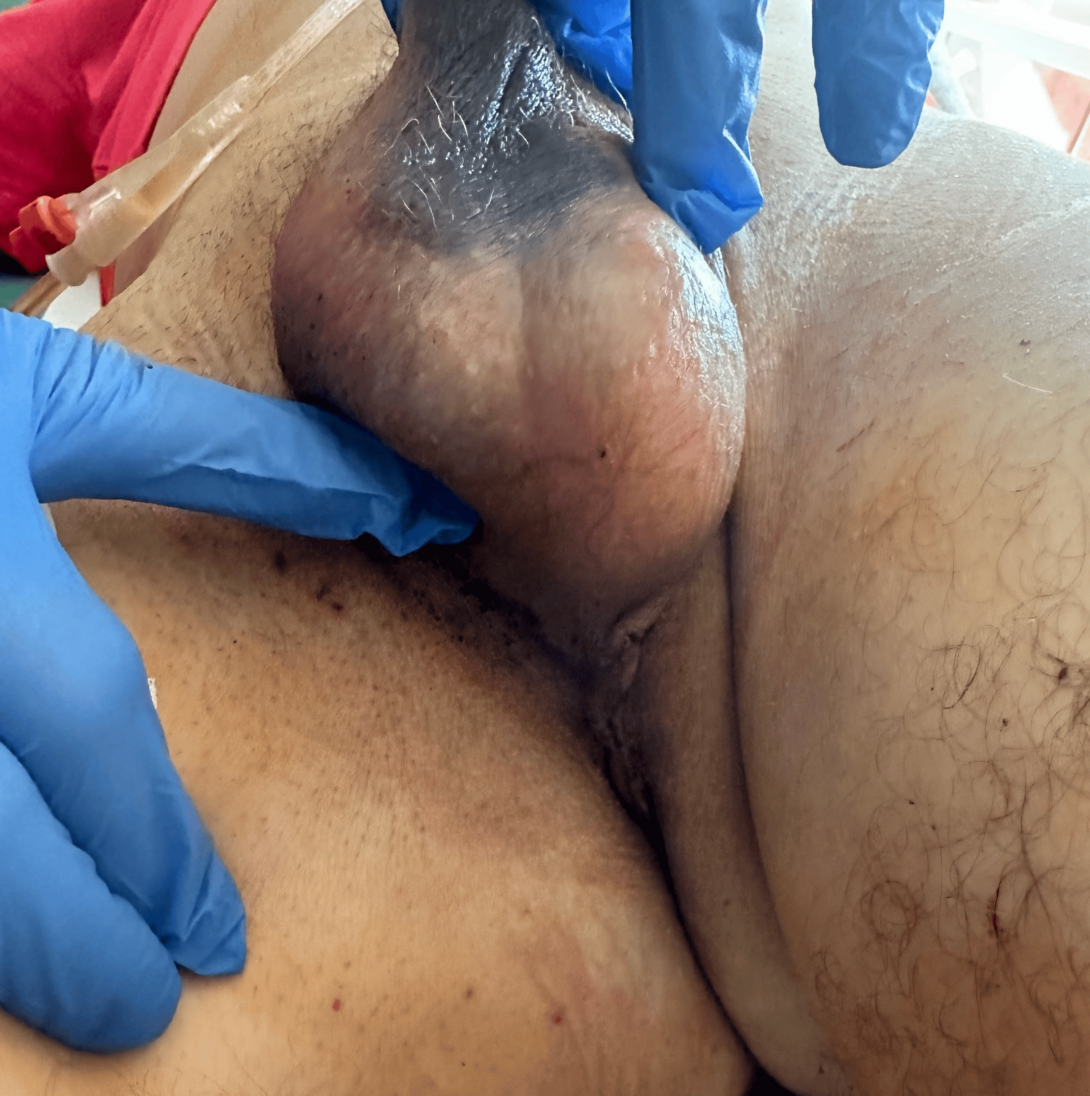
Perineal incision on postoperative day 2 demonstrating proper wound healing without hematoma

**Figure 5:**
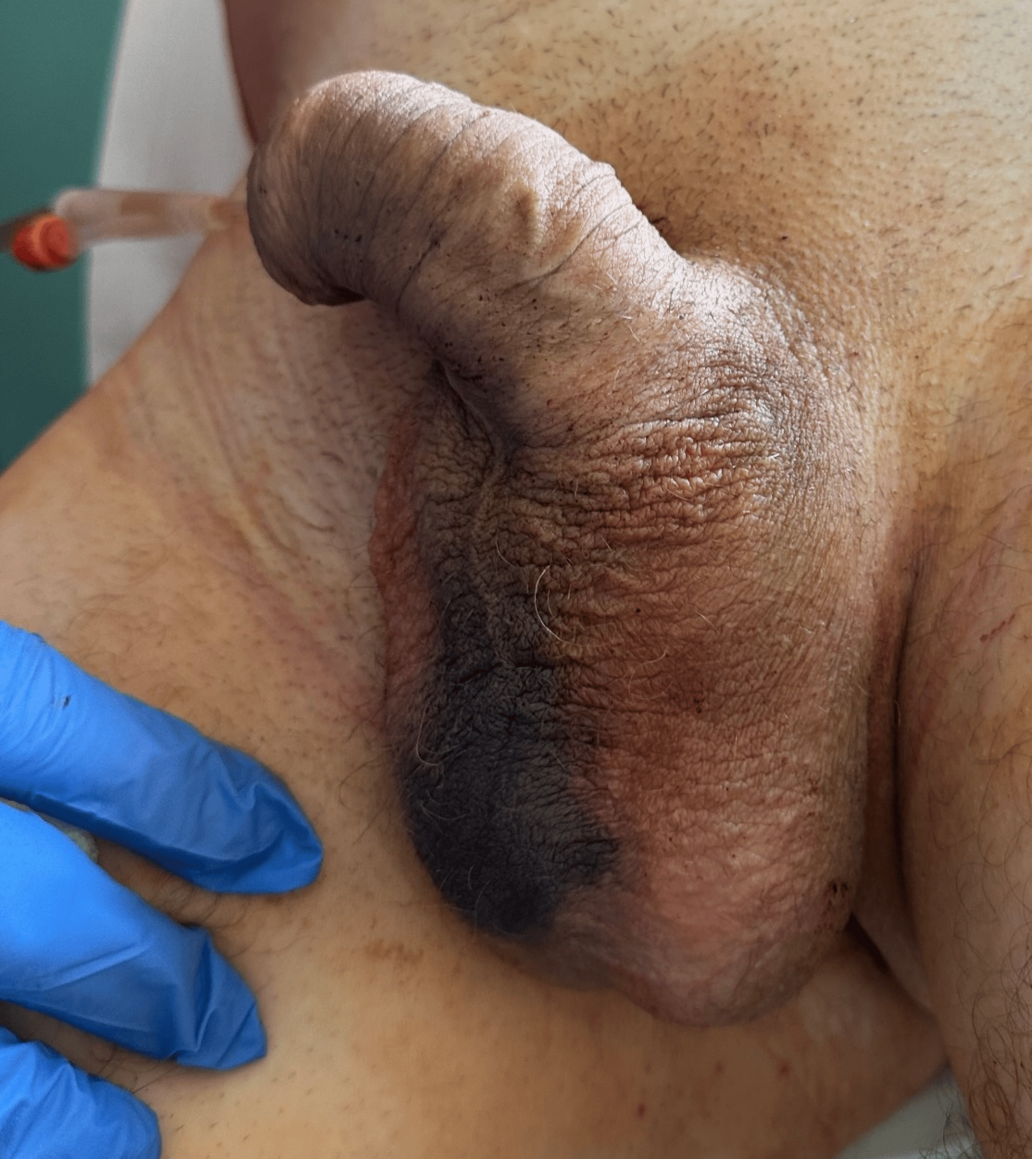
Scrotal/perineal view on postoperative day 2 with minor ecchymosis; catheter and dressing in place

By postoperative day 2, the patient was ready for discharge. Perineal inspection showed well-healed tissue without hematoma or signs of infection; scrotal/perineal ecchymosis was minimal, the suprapubic catheter remained correctly positioned, and the dressing was intact (Figures 4, 5). The patient reported complete relief of urinary retention and had no pain or systemic symptoms. He was discharged with clear instructions regarding warning signs, such as fever, hematuria, increasing pain, or catheter malfunction, and was scheduled to return for Foley catheter removal after 14 days. Routine postoperative uroflowmetry performed six weeks after catheter removal demonstrated a satisfactory maximum flow rate (Qmax) of 17 mL/s with no significant postvoid residual volume, indicating a good functional outcome. The patient reported normal voiding with no urinary retention, straining, or incontinence during follow-up.

## Discussion

Bulbar urethral strictures, particularly those involving long segments with complete obliteration, represent a significant reconstructive challenge. In the present case, combined retrograde and antegrade urethrography confirmed a 3-5 cm segmental obliteration of the bulbar urethra, necessitating formal open reconstruction. This diagnostic strategy is essential in accurately defining the location and length of the stricture, which guides the surgical approach [1,2]. According to the American Urological Association (AUA) guidelines, endoscopic management such as dilation or direct visual internal urethrotomy (DVIU) is unlikely to be effective in strictures longer than 2 cm or in cases with complete obliteration [3]. Therefore, open urethroplasty remains the treatment of choice in such scenarios. Comparative studies have demonstrated that patients with traumatic bulbar strictures longer than 2 cm have poorer outcomes with repeated endoscopic procedures compared to primary open urethroplasty [2-4]. For appropriately selected cases, anastomotic (end-to-end) urethroplasty is indicated and can be successfully performed for strictures up to 3-5 cm with reported success rates exceeding 85-90% [2,4]. The key principles include tension-free anastomosis and preservation of the surrounding vascular supply to minimize ischemic complications. Longer strictures or those involving panurethral segments may require substitution urethroplasty using grafts (e.g., buccal mucosa) or flaps [4,5]. In the presented case, the location and length of the stricture allowed for successful end-to-end reconstruction without the need for tissue substitution. Finally, surgeon experience and stricture characteristics such as etiology (trauma vs. iatrogenic), degree of spongiofibrosis, and previous interventions are all known to influence outcomes [5]. Proper patient selection and meticulous technique are crucial to optimize functional recovery and minimize the risk of recurrence.

## Conclusions

This case highlights the successful management of a challenging, long-segment bulbar urethral stricture resulting from pelvic trauma, achieved through a perineal end-to-end urethroplasty. Accurate delineation of the stricture using antegrade urethrography was pivotal in planning the procedure and ensuring an appropriate surgical strategy. The utilization of tension-free anastomosis over a 16 Ch catheter with interrupted 5‑0 Monocryl sutures, combined with evidence-based perioperative care, namely, preoperative gentamicin prophylaxis, thromboprophylaxis with enoxaparin, extended catheter maintenance, and postoperative antibiotic cover, contributed to an uncomplicated recovery and well-healed surgical site. This case reinforces that, in selected patients, even extensive strictures can be effectively managed with primary anastomotic reconstruction, provided that meticulous surgical technique is paired with comprehensive perioperative management and close follow-up. By sharing this example, we aim to support clinicians in optimizing care for complex urethral strictures and demonstrating that a structured, stepwise approach can lead to excellent functional outcomes.
